# Global Health Education: International Collaboration at ICDDR,B

**DOI:** 10.3329/jhpn.v28i6.6600

**Published:** 2010-12

**Authors:** Omar A. Khan, Mark Pietroni, Alejandro Cravioto

**Affiliations:** ^1^ Department of Family Medicine, University of Vermont College of Medicine, Burlington, Vermont 05405, USA; ^2^ ICDDR,B, GPO Box 128, Dhaka 1000, Bangladesh

**Keywords:** Collaborations, Health education, Public health

## Abstract

The purpose of this commentary is to provide an overview of the growing interest in global health education at ICDDR,B and to review examples of how this has grown from public-health research and education to include clinical education (medical and nursing) as well. This parallels the growth of the institution, with an increased focus on educational linkages within and beyond Bangladesh and the rise in interest in global health at western medical schools. Specific collaborations, their setup and structure are described. This is presented as a model for other centres of excellence in developing countries to engage their partners in the South and North on matters of education and research for mutual cooperation and benefit.

## INTRODUCTION

The global nature of public health has been recognized for decades, if not centuries. ICDDR,B, based in Dhaka, Bangladesh, has played a significant role in research and innovation in the global public-health arena, historically in the areas of child health and diarrhoeal diseases, and more recently in broader care, including maternal and child health and HIV/AIDS. There has, in recent years, been an increasing emphasis on clinical care delivered at the institution as well, primarily through the main hospital. To parallel this change, there is unprecedented interest in clinical global health at U.S. medical schools and for the desire to seek international medical electives in the developing-world setting.

Most U.S. physicians will either serve international patients through travel-related medical needs and/or work directly with those who do not reside permanently within the country (1). Coupled with increasing globalization, this presents an opportunity for clinical practice outside one's borders—both geographical and the boundaries of traditional medical training, which typically deal with the diseases of the surrounding community. Meanwhile, interest in global health among U.S. medical students has increased dramatically. The percentage of medical students participating in international electives has increased from 6.4% in 1984 (2) to 23.1% in 2007 (1). Due to expanding undergraduate opportunities, matriculating medical students increasingly have prior international experience, and 20–30% of medical students go overseas (3). Of 116 United States allopathic schools surveyed in 2010, 79 had active student interest groups pertaining to global or international health (4).

According to the Consortium for Universities in Global Health, “[t]he last 20 years has seen an unprecedented interest in global health among faculty and students in North American universities. The response of universities could not keep pace with the level of enthusiasm and demand …” (5).

ICDDR,B hosts a number of well-established programmes, including Fogarty scholarship recipients, which bring students from the USA and other countries to conduct research for Masters and PhD programmes, usually in collaboration with institutions in the students' home countries. However, until recently, there have been no similar programmes in clinical medicine.

## RECENT DEVELOPMENTS IN EDUCATIONAL COLLABORATION

As the institution has increased in size and scope, so have its educational collaborations in the country and the region. A significant change has come about in engaging those at the training phase of their career. As an example, a linkage with the Bangladesh-based James P. Grant School of Public Health of BRAC University has been formalized, and the ICDDR,B's Executive Director serves a dual role as the School's Associate Dean. This enables the institution's staff to teach courses in their area of expertise at the School, in areas such as epidemiology, clinical trials, and health policy. In this sense, the partnership mirrors that of the U.S. Centers for Disease Control and Prevention and nearby Emory University. Public-health practitioners, by taking on a faculty role, remain engaged in an intellectual activity while engaging with learners and faculty members in an academic environment. Clearly, the students benefit greatly by learning from those in practice.

Within its hospital, ICDDR,B has recently established formal residency-style training programmes for its junior medical and nursing staff with the aim of improving the quality of clinical care and growing ‘in-house’ the next generation of senior staff. Such programmes are unique in Bangladesh and now take their place alongside the institution's equally unique research training activities.

Simultaneously, formal and informal arrangements with international universities have been developed. One such example is the close collaboration with an American university, i.e. the University of Vermont College of Medicine (UVM). Faculty members at UVM were familiar with the work of ICDDR,B through their own training and work there. As such, the institution was an ideal location to form a relationship with due to its presence in the capital city of Dhaka; the clinical volume available; the opportunities for mentorship and teaching; and language not being a significant barrier.

The relationship between ICDDR,B and UVM was formalized via a Memorandum of Understanding (MoU). The application process is formalized as well and consists of the potential trainees completing the standard ICDDR,B application for short-term training. Faculty members at both institutions review the applicants and select the ones most likely to be prepared for and to benefit from the experience; 3–4 students a year are selected for a one-month clinical elective. Trainees are usually at the end of their medical school education and about to enter residency training, usually in family medicine or paediatrics. Pre-rotation preparation, education, and logistics are coordinated by the global health curriculum director at UVM while in-country arrangements are organized by the Student Welfare Officer at ICDDR,B.

## OPPORTUNITIES

In general, the collaboration on clinical education has worked well. As one example of a collaboration between a centre of excellence in a developing world (ICDDR,B) and a western university (UVM), the initial preparation and formalization were conducted in a thoughtful and transparent manner. Much of the success can thus be attributed to (a) process factors and (b) personnel factors.

In terms of process, having a uniform and standard MoU at the outset is useful and establishes trust. Mutual agreement on selection criteria for students and the roles and responsibilities of each institution need to be clarified well before the first student applies. Similarly, the application process requires coordination between the host institute's (ICDDR,B's) training or education division, counterpart directors at either end, student coordinator at the host centre, and the applicant. We have found a standard pre-elective checklist to be very helpful, and this has, in fact, been refined and modified with the experience of each successive student.

In terms of personnel, establishing a working relationship between the counterpart faculty and course directors is essential. ICDDR,B has been a home, at some point or another, to a large cross-section of those working in global health, wherever they may currently be based. As such, these relationships can be harnessed for maximum effect for the benefit of educational programme development. In the case above, the key personnel involved are in touch with all critical points relating to students' participation. This carries over into the post-elective evaluation where a candid appraisal of the experience, from the mentor's and the student's perspective, is important for maintaining high quality and continuous programme improvement.

Potential issues and barriers should make themselves visible to be solved quickly. As an example, until ICDDR,B had an on-site student coordinator, this role was handled by the counterpart directors, individual preceptors, and training division personnel. In reality, the presence of a student coordinator is one we would highly recommend. This individual serves as a pre-arrival liaison and a critical coordinator of the experience once the student starts. Issues addressed can include housing, where to meet for rounds, and what research opportunities might exist. This tends to make the experience a great deal more uniform across students; it also can identify issues before they arise.

In any collaboration involving trainees in a developing setting, security concerns inevitably arise. ICDDR,B has the good fortune to be located in an area of relative peace and calm; certainly no worse than any megacity in the developed world. It also hosts many western expatriates, working and living alongside their Bangladesh-based colleagues. This environment presents inherent advantages and has helped sustain trainees' interest despite conflicts elsewhere in the South Asia region. In our experience, the combination of an excellent professional experience and a well-coordinated programme are key to an overall successful rotation; this has been observed elsewhere as well (6, 7).

## FUTURE DIRECTIONS

Building educational collaborations in clinical care has been an important step for an institution classically steeped in a research tradition. It has also facilitated the global health education of successive cohorts of medical students from the University of Vermont and elsewhere. This collaboration has allowed ICDDR,B to expand a ‘culture of education’ in clinical staff, which remains a work in progress. At the same time, while the majority of work at ICDDR,B may remain in research activities, it is likely that this will, in the near future, more equally balance the classic academic tripod of research, teaching, and service (i.e. clinical work).

The advancement of the size and scope of the ICDDR,B hospital have facilitated medical educational opportunities mentioned above. An additional and important area is nursing, a critical health profession which Bangladesh is in short supply of. The institution has established a similar international link with the Faculty of Nursing Education at Trondheim University, Norway, under which around 10 nursing students spend a month at ICDDR,B each year.

From the developed-country perspective, increasing global engagement means a greater emphasis on global health education and service (8). This has positive implications for further intellectual and financial support of such initiatives, which are likely to grow in the near term (9).

## CONCLUSION

Educational collaborations, whether South-South or North-South, hold great potential for all parties involved. This commentary highlights some points of importance which made this collaboration succeed. It also suggests that no one area progresses in isolation: as ICDDR,B's mandate has grown overall, so have its interests in education and clinical care grown, along with its historic excellence in research. This has fortuitously paralleled the interest at UVM and elsewhere in global health. Thus, the formation of such collaborations can actually serve as a lens through which to view such progress as a whole, to the mutual benefit of the partner institutions, the trainees, and ultimately the communities they serve.

## ACKNOWLEDGEMENTS

The authors thank Dr. Beth Kirkpatrick, University of Vermont College of Medicine, USA, for her valuable comments on a draft of this manuscript.
